# Single cell analysis of docosahexaenoic acid suppression of sequential LPS-induced proinflammatory and interferon-regulated gene expression in the macrophage

**DOI:** 10.3389/fimmu.2022.993614

**Published:** 2022-11-03

**Authors:** Kathryn A. Wierenga, Frank M. Riemers, Bart Westendorp, Jack R. Harkema, James J. Pestka

**Affiliations:** ^1^ Department of Biochemistry and Molecular Biology, Michigan State University, Lansing, MI, United States; ^2^ Institute for Integrative Toxicology, Michigan State University, Lansing, MI, United States; ^3^ Department of Biomolecular Health Sciences, Faculty of Veterinary Medicine, Utrecht University, Utrecht, Netherlands; ^4^ Department of Pathobiology and Diagnostic Investigation, Michigan State University, Lansing, MI, United States; ^5^ Department of Microbiology and Molecular Genetics, Michigan State University, Lansing, MI, United States; ^6^ Department of Food Science and Human Nutrition, Michigan State University, Lansing, MI, United States

**Keywords:** inflammatory gene expression, omega-3 polyunsaturated fatty acids, cholesterol metabolism, TLR4 signaling, NF-κB, IFN signaling, macrophage, scRNAseq

## Abstract

Preclinical and clinical studies suggest that consumption of long chain omega-3 polyunsaturated fatty acids (PUFAs) reduces severity of chronic inflammatory and autoimmune diseases. While these ameliorative effects are conventionally associated with downregulated expression of proinflammatory cytokine and chemokine genes, our laboratory has recently identified Type 1 interferon (IFN1)-regulated gene expression to be another key target of omega-3 PUFAs. Here we used single cell RNA sequencing (scRNAseq) to gain new mechanistic perspectives on how the omega-3 PUFA docosahexaenoic acid (DHA) influences TLR4-driven proinflammatory and IFN1-regulated gene expression in a novel self-renewing murine fetal liver-derived macrophage (FLM) model. FLMs were cultured with 25 µM DHA or vehicle for 24 h, treated with modest concentration of LPS (20 ng/ml) for 1 and 4 h, and then subjected to scRNAseq using the 10X Chromium System. At 0 h (i.e., in the absence of LPS), DHA increased expression of genes associated with the NRF2 antioxidant response (e.g. *Sqstm1*, *Hmox1, Chchd10*) and metal homeostasis (e.g.*Mt1*, *Mt2, Ftl1, Fth1*), both of which are consistent with DHA-induced polarization of FLMs to a more anti-inflammatory phenotype. At 1 h post-LPS treatment, DHA inhibited LPS-induced cholesterol synthesis genes (e.g. *Scd1, Scd2*, *Pmvk, Cyp51, Hmgcs1*, and *Fdps)* which potentially could contribute to interference with TLR4-mediated inflammatory signaling. At 4 h post-LPS treatment, LPS-treated FLMs reflected a more robust inflammatory response including upregulation of proinflammatory cytokine (e.g. *Il1a, Il1b, Tnf*) and chemokine (e.g.*Ccl2, Ccl3, Ccl4, Ccl7*) genes as well as IFN1-regulated genes (e.g. *Irf7, Mx1, Oasl1, Ifit1*), many of which were suppressed by DHA. Using single-cell regulatory network inference and clustering (SCENIC) to identify gene expression networks, we found DHA modestly downregulated LPS-induced expression of NF-κB-target genes. Importantly, LPS induced a subset of FLMs simultaneously expressing NF-κB- and IRF7/STAT1/STAT2-target genes that were conspicuously absent in DHA-pretreated FLMs. Thus, DHA potently targeted both the NF-κB and the IFN1 responses. Altogether, scRNAseq generated a valuable dataset that provides new insights into multiple overlapping mechanisms by which DHA may transcriptionally or post-transcriptionally regulate LPS-induced proinflammatory and IFN1-driven responses in macrophages.

## Introduction

Macrophages, a highly plastic innate myeloid immune cell population, can be polarized to a range of phenotypes that promote or resolve inflammation (1). Activation of toll-like receptors (TLRs) and cytokine receptors skews macrophages towards a more proinflammatory phenotype (e.g. “M1”) that is commonly observed in chronic inflammatory and autoimmune conditions, such as atherosclerosis (2), obesity (3), rheumatoid arthritis (4), and lupus (5). Contrastingly, skewing macrophages toward a proresolving phenotypes (e.g. “M2”) can initiate repair of damaged tissue, phagocytose and clear dead cells, and suppress the inflammatory response (1). Thus, interventions that suppress macrophage activation or promote their proresolving effects may be able to alleviate chronic inflammation and autoimmune diseases.

Pathogen-derived stimuli are frequently used to activate immune cells to investigate their inflammatory response *in vitro* and *in vivo*. Bacterial lipopolysaccharide (LPS), a prototypical pathogen-derived inflammatory trigger, is a component of the gram-negative bacterial cell wall and a potent agonist for the pattern recognition receptor TLR4 in macrophages (6). TLR4 promotes classical activation to the M1 phenotype *via* multiple signaling pathways that involve MAP kinases, NF-κB, and interferon (IFN) response factors (IRF) that promote time-dependent upregulation of inflammation-linked genes (7, 8). Genes associated with transcriptional machinery are among the first induced because of TLR4 activation, followed by a large repertoire of proinflammatory cytokine, chemokine, and Type 1 interferon (IFN1)-regulated genes. In the absence of prolonged stimulation, TLR4-stimulated inflammation is modest and self-resolving. Thus, induced genes and their protein products peak at specific timepoints, followed by rapid degradation. However, in situations of prolonged and unresolved inflammation, many of these genes may remain elevated, contributing to pathophysiological effects (9, 10). Chronic inflammatory and autoimmune diseases often result from such aberrantly prolonged signaling, making it of paramount importance to identify ways to subdue proinflammatory and IFN1-regulated gene responses and/or hasten their resolution.

Preclinical and clinical studies reveal that dietary or pharmacological intervention with long chain omega-3 polyunsaturated fatty acids (PUFAs) such as docosahexaenoic acid (DHA) and eicosapentaenoic acid (EPA) ameliorate symptoms and reduce biomarkers in inflammatory diseases, including rheumatoid arthritis (11), cardiovascular disease (12, 13), and lupus (14, 15). *In vivo*, dietary supplementation with omega-3 PUFAs elevates their presence in the phospholipid membranes in individual tissues, red blood cells, and blood lipid pools. There are multiple macrophage-dependent mechanisms by which elevated omega-3 PUFAs may suppress inflammation and promote resolution [reviewed in (16)]. For example, increasing membrane omega-3 PUFAs can influence lipid raft formation, altering aggregation and activation of transmembrane receptors that initiate inflammatory signaling pathways. Another mechanism is interference with NF-κB signaling by intracellular activation of the transcription factor PPARγ or extracellular activation G-coupled protein receptor (GPCRs) such as GPR40 and GPR120. Finally, shifting the balance of lipid metabolites from pro-inflammatory omega-6-derived oxylipins to proresolving omega-3-derived oxylipins, such as the highly bioactive resolvins, maresins, and protectins, promotes an anti-inflammatory phenotype (17).

While investigations employing supplementation of macrophages *in vitro* with long chain omega-3 PUFAs have provided insight into how these dietary lipids influence inflammatory pathways (16, 18, 19), these mechanistic studies typically have relied on bulk mRNA analyses and often utilized cloned macrophage cell lines. The rationale for this investigation, was to address these limitations by employing single cell RNA sequencing (scRNAseq) in conjunction with a novel, self-renewing fetal liver-derived macrophage (FLM) model (20) to better understand how DHA influences LPS-triggered inflammation-related gene expression of individual macrophages. Our findings yield new perspectives into multiple overlapping mechanisms by which DHA may influence time-dependent TLR4-driven proinflammatory and IFN1-regulated gene responses in macrophages.

## Methods

### Animals and euthanasia

Experimental protocols were approved by the Institutional Animal Care and Use Committee at MSU (AUF #PROTO201800113). C57BL/6J mice (strain 000664) were obtained from The Jackson Laboratory (Bar Harbor, ME). Mice were given free access to food and water under controlled conditions (humidity: 40–55%; lighting: 12-h light/dark cycles; and temperature: 24 ± 2°C) (21). Mice were bred and livers were excised from murine fetuses at 14-18 gestational days (20). Dams were euthanized by CO_2_ inhalation for 10 min to ensure death to neonates, which are resistant to anoxia. Cervical dislocation was used as a secondary form of death for the dam. Fetuses were immediately removed, and loss of maternal blood supply served as a secondary form of death for the fetuses.

### Generation and maintenance of FLM cultures

FLMs were generated and maintained using a modification of the method described by Fejer and coworkers (20). Briefly, fetal livers were dissociated into a single cell suspension in sterile phosphate buffered saline (PBS), filtered through a 70-micron filter, and centrifuged at 220 *x*g for 5 minutes. Cells were washed twice with sterile PBS, resuspended in modified Roswell Park Memorial Institute (mRPMI) Medium (Thermo Fisher) containing 10% fetal bovine serum (FBS, Thermo Fisher), 1% penicillin-streptomycin (P/S, Thermo Fisher) and 30 ng/mL murine granulocyte-monocyte colony stimulating factor (mGM-CSF, Peprotech), and plated in 10-cm treated culture dishes (1 liver/dish). The following day, half of the media was replaced with fresh mRPMI. Media was refreshed in this manner every 2 to 3 d, until an adherent monolayer was achieved within 1 to 2 wk. At this time, cells were either frozen for cryostorage or passaged for experiments. Initially, FLMs cultured under these conditions have an ovoid morphology and proliferate slowly, but after approximately 5 to 10 passages, cells become spindleloid and proliferate rapidly. FLMs used for experiments in this study were between passage 10 and 20.

For fatty acid incorporation studies, DHA (NuChek Prep, Elysian, MN) was prepared as a 3:1 complex with fatty-acid free bovine serum albumin (BSA, Millipore Sigma, Burlington, MA) (18, 22). Cells were then incubated for 24 h in serum-reduced mRPMI medium (0.25% FBS, 1% P/S, 30 ng/mL mGM-CSF) with 25 µM DHA + 8.3 µM BSA or with 8.3 µM BSA as vehicle. The serum concentration was reduced to maximize DHA’s incorporation into the phospholipid membrane by removing competing fatty acids present in serum.

For TLR4 stimulation, a stock solution of LPS (*Salmonella enterica* serotype typhimurium containing <1% protein impurities, Millipore Sigma) was prepared in sterile PBS. The solution was thoroughly vortexed and sonicated before use, and dilutions were prepared in serum-reduced mRPMI medium. Cells were stimulated with 20 ng/mL LPS, which was chosen based on a time course and dose response that showed sufficient expression of a few key inflammatory genes with this dose of LPS at 1 and 4 h (**Figure S1**).

### Single cell isolation, library preparation, and sequencing

**Figure 1:**
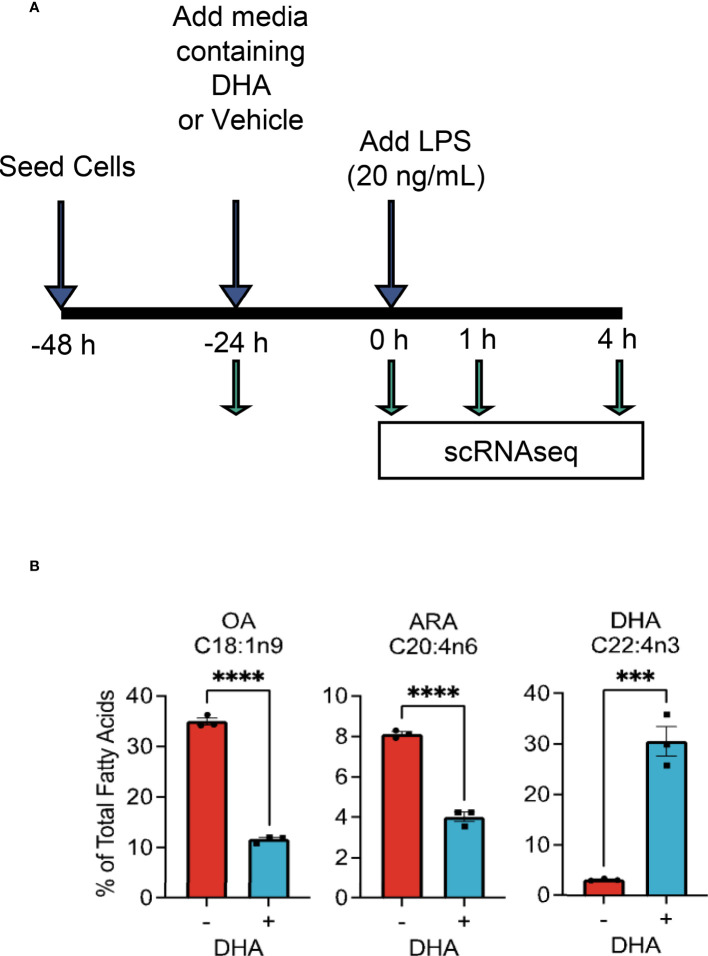
Experimental design for scRNAseq and DHA incorporation into FLMs. **(A)** FLMs were seeded in 6 well plates at -48 h in mRPMI medium containing 10% FBS. At -24h, medium was replaced with mRPMI containing 0.25% FBS with either DHA or Veh. At time 0 h (24h after DHA supplementation), cells were treated with 20 ng/mL LPS. Sample start times were staggered to allow collection of all samples simultaneously, after the indicated treatment times. **(B)** DHA supplementation results in increased DHA levels in the phospholipid membrane at the expense of oleic acid and arachidonic acid. Cells treated with DHA or Veh for 24 h were collected in methanol and analyzed by GC-FID at OmegaQuant LLC. Fatty acid levels expressed as a percent of all fatty acids measured. Asterisks indicate significant differences (****p<0.0001, ***p<0.001) between Veh (-) and DHA (+) treatment groups, as assessed by Student’s t-tests.

The overall experimental design for deriving scRNAseq samples is depicted in **Figure 1A**. Briefly, FLMs were seeded in 6-well plates at -48 h in complete media. At -24 h, media was changed to contain 25 μM DHA or vehicle (Veh, 8.3 μM BSA) and 0.25% FBS. At time 0 h (24 h after DHA supplementation), cells were treated with 20 ng/mL LPS. Treatment conditions were as follows: i) Con (control, collected at -24 h); ii) Veh.LPS.0 (treated with 8.3 μM BSA, collected at 0 h); iii) DHA.LPS.0 (treated with 25 μM DHA + 8.3 μM BSA, collected at 0 h); iv) Veh.LPS.1 (treated with 8.3 μM BSA, collected 1 h post-LPS); v) DHA.LPS.1 (treated with 25 μM DHA + 8.3 μM BSA, collected at 1 h post-LPS); vi) Veh.LPS.4 (treated with 8.3 μM BSA, collected 4 h post-LPS), and vii) DHA.LPS.4 (treated with 25 μM DHA + 8.3 μM BSA, collected at 4 h post-LPS). Sample start times were staggered to allow collection of all samples simultaneously, after the indicated treatment times. Cells were lifted from 6-well plates using Accutase^®^ (Millipore Sigma) and washed twice with ice cold sterile PBS to remove residual enzyme. Cell viability immediately after lifting and prior to single cell isolation was assessed by Trypan Blue exclusion test. All treatment groups had a viability of >90%. Single cells were isolated and RNA libraries prepared using the 10x Chromium SingleCell 3′ RNAseq kit (v3 Chemistry, 10X Genomics), per the manufacturer’s instructions. Sample quality control was performed at the Michigan State University Genomics Core followed by sequencing at Novogene. Libraries were sequenced on an Illumina Novaseq using S4 chemistry, obtaining >100K reads/cell. Reads were demultiplexed and subsequently counted with the Cell Ranger v2.1.1 (23) mkfastq and count pipeline respectively.

### Seurat analysis

Data were analyzed further with Seurat package v3.1.1 (24) in RStudio-server v1.3.959-1 (25) using R v3.06 (26). After importing the raw counts they were filtered to remove low quality cells (**Figures S2A, B**). Only cells with fewer than 20% mitochondrial genes and over 20000 unique UMI reads mapping to at least 2000 unique genes were included. The remaining cells were then subjected to normalization with the Seurat SCTransform function. Subsequently, the dimensionality of the data was reduced using a Principal Component Analysis (PCA). Clusters were identified using the FindClusters function in Seurat, which were visualized using the Uniform Manifold Approximation and Projection (UMAP) technique.

### SCENIC analysis

Single-cell regulatory network inference and clustering (SCENIC) was used to detect which transcription factor programs were most strongly affected by DHA in 4 h LPS-treated cells (27). Briefly, the SCENIC workflow consists of three steps: 1) the GENIE3 package is used to identify genes co-expressed with transcription factors, 2) the RcisTarget package is used to perform *cis*-regulatory motif analyses of co-expressed genes to identify potential binding targets for the transcription factor in question, and 3) the AUCell package is used to assign a score to each cell based on the activity of the regulon, which is the network of genes likely driven by an individual transcription factor.

### Heatmaps

Heatmaps were generated using the pheatmap package in R Studio (28), based on scaled gene expression data or regulon AUC values for individual cells. Ward’s criteria were used as the clustering method. The “cutree_columns” function was used to break the heatmap into clusters. No scaling was applied as the input values were already scaled for individual cells and regulons.

### Bulk qPCR

For confirmatory bulk PCR studies, cells were treated in 12-well plates with 25 μM DHA/8.3 μM BSA or Veh (8.3 μM BSA) for 24 h and 20 ng/mL LPS for 1 and 4 h. Approximately 5x10^5^ cells were collected for RNA extraction and gene expression analysis carried out as previously described (18, 29). Gene expression was calculated as the relative copy number (18, 30).

### Western blotting

NF-κB Western blotting was carried out as reported previously (18). Briefly, whole cell lysates were isolated, with Halt™ Protease and Phosphatase inhibitors (Thermo Fisher) included in RIPA buffer (Thermo Fisher) during lysis. Nuclear and cytoplasmic extracts were prepared using a Nuclear Extract kit (Active Motif). Protein was quantified using a Pierce™ BCA protein assay (Thermo Fisher), after which all samples were adjusted to the same concentration. Samples were loaded in lanes of pre-cast 4-20% Mini-Protean TGX Protein Gels (Bio-Rad) and electrophoresis performed at 100V for 90 min in a BioRad mini-PROTEAN tetra vertical electrophoresis chamber. Proteins were transferred to a low fluorescence nitrocellulose membrane (Bio-Rad) using the Bio-Rad TransBlot Turbo System, per the manufacturer’s instructions. Antibody binding was performed using an iBind Flex apparatus per the manufacturer’s instructions. The following primary antibodies used at the indicated dilution: Rabbit anti-Actin (Cell Signaling Technologies, 1:4000), NF-κB (CST, 1:1000), phospho-NF-κB (CST, 1:1000), IκB (CST, 1:1000), phospho-IKB kinase (CST, 1:1000). The following Licor (Lincoln, Nebraska) near infrared fluorescent secondary antibodies were used: IRDye^®^ 680LT (1:4000) and IRDye^®^ 800CW (1:3000). Blots were read using a Licor Odyssey Imaging System.

### Fatty acid analyses

To measure phospholipid fatty acid content, cell pellets were stored in 100% methanol at -80°C until analysis by gas chromatography (GC) with flame ionization detection at OmegaQuant, LLC. Cell pellets were transferred to screw-cap glass vials containing 1,2-ditricosanoyl-sn-glycero-3-phosphocholine (di-C23:0 PL) (Avanti Polar Lipids, USA) as an internal standard followed by a modified Folch extraction. A portion of the organic layer was spotted on a TLC plate developed with 8:2:0.15 (hexane:ethyl ether:acidic acid) to separate the lipid fractions. After the TLC plate was dry the phospholipid band was scrapped into a screw-cap glass vial with methanol containing 14% boron trifluoride (Sigma-Aldrich, St. Louis, MO). The vial was briefly vortexed and heated to 100˚C for 10 minutes. After cooling, HPLC grade water and hexane (EMD Chemicals, USA) were added sequentially, the tubes were recapped, vortexed and centrifuged promote phase separation. GC of the hexane layer was carried out using a GC2010 Gas Chromatograph (Shimadzu Corporation, Columbia, MD) equipped with a SP2560, 100-m fused silica capillary column (0.25 mm internal diameter, 0.2 μm film thickness; Supelco, Bellefonte, PA).

Fatty acids were identified by comparison with a standard mixture of fatty acids (GLC 782, NuCheck Prep) and an internal standard (C23:0 FAME, NuCheck Prep). The di-C23:0 PL was used to calculate recovery efficiency of the assay and applied to all fatty acids. The following 24 fatty acids (by class) were identified: saturated (14:0, 16:0, 18:0, 20:0, 22:0 24:0); cis monounsaturated (16:1, 18:1, 20:1, 24:1); trans (16:1, 18:1, 18:2), cis n-6 polyunsaturated (18:2, 18:3, 20:2, 20:3, 20:4, 22:4, 22:5); cis n-3 polyunsaturated (18:3, 20:5, 22:5, 22:6). Fatty acid composition was expressed as a percent of total identified fatty acids and concentrations as µg/vial of cell pellets.

### Data visualization and statistics

Data were plotted in R Studio version 1.1.442 using ggplot2 (31) and in GraphPad Prism version 9.1.0 (San Diego, California, USA, www.graphpad.com). Non-parametric versions of statistical tests were used where appropriate, as noted in figure legends.

## Results and discussion

### DHA supplementation increases DHA in FLM membrane

We selected the dose of 25 µM DHA based on previous findings in RAW264.7 murine macrophages, where this dose effectively protected these cells from cell death and IL-1 cytokine release following NLRP3 inflammasome activation (18). This fatty acid supplementation protocol resulted in a 10-fold increase incorporation of DHA into the phospholipid fraction of FLMs at 0 h before LPS activation with concurrent reductions of oleic acid, a monounsaturated fatty acid, and arachidonic acid, an omega-6 PUFA (**Figure 1B** and **Table 1**). These results are consistent with our previous findings (18), suggesting similar incorporation efficiency in RAW264.7 macrophages and self-renewing FLMs.

**Table 1:** Fatty acid composition of cells treated with Veh (BSA, 8.3 μM) or DHA-BSA complex (25 μM DHA, 8.3 μM BSA).

		Veh	DHA
Fatty acid	Chemical formula	*% of total fatty acids, mean± SD*	
Myristic	C14:0	2.18 ± 0.19	3.23 ± 0.43*
Palmitic	C16:0	10.76 ± 0.7	19.54 ± 5.32*
Palmitelaidic	C16:1ω7t	0.73 ± 0.34	0.57 ± 0.29
Palmitoleic	C16:1ω7	4.39 ± 0.64	0.77 ± 0.31***
Stearic	C18:0	12.85 ± 0.47	13.54 ± 3.75
Elaidic	C18:1t	1.64 ± 0.07	0.66 ± 0.64
Oleic	C18:1ω9	34.97 ± 1.17	11.68 ± 0.66***
Linolaidic	C18:2ω6t	1.68 ± 0.81	1.78 ± 0.48
Linoleic	C18:2ω6	3.10 ± 0.56	1.95 ± 0.32*
Arachidic	C20:0	0.47 ± 0.16	0.63 ± 0.20
Gamma-Linolenic	C18:3ω6	0.48 ± 0.15	0.30 ± 0.04
9-Eicosanoic	C20:1ω9	2.32 ± 0.21	1.25 ± 0.34**
Alpha-Linolenic	C18:3ω3	0.35 ± 0.04	0.29 ± 0.09
Eicosadienoic	C20:2ω6	0.28 ± 0.19	0.38 ± 0.18
Behenic	C22:0	1.23 ± 0.41	1.01 ± 0.22
DGLA	C20:3ω6	0.72 ± 0.21	1.14 ± 0.39
Arachidonic	C20:4ω6	8.13 ± 0.19	4.03 ± 0.40***
Lignoceric	C24:0	1.04 ± 0.34	0.71 ± 0.22
Eicosapentaenoic	C20:5ω3	0.73 ± 0.18	1.17 ± 0.35
Nervonic	C24:1ω9	0.58 ± 0.21	0.52 ± 0.32
Docosatetraenoic	C22:4ω6	3.36 ± 0.36	1.48 ± 0.22**
Docosapentaenoic-ω6	C22:5ω6	1.59 ± 0.16	0.56 ± 0.40*
Docosapentaenoic-ω6	C22:5ω3	3.19 ± 0.09	2.30 ± 0.41*
Docosahexaenoic	C22:6ω3	3.20 ± 0.21	30.53 ± 5.09***
Total SFA		28.54 ± 1.22	38.66 ± 9.08
Total MUFA		44.64 ± 1.70	15.45 ± 2.10***
Total ω-6		19.35 ± 0.66	11.60 ± 1.68**
Total ω-3		6.74 ± 0.20	33.11 ± 5.45**
Omega-3 Index		3.93 ± 0.37	31.70 ± 5.15***

Asterisks indicate significant change in DHA-supplemented vs Veh group, as determined by Student’s t-test (*p<0.05, **p<0.01, ***p<0.001).

### Clustering is driven by LPS treatment and cell cycle phase

Seurat identified ten distinct clusters of cells, which visually separated into four time-associated groups (**Figure S3A**) When individual cells were colored according to treatment group, it was evident that culturing for 24 h with reduced serum resulted in a separation from the control group at 0 h (**Figure S3B**). Cells treated with and without LPS for 1 or 4 h separated from one another in the UMAP projection. Veh- and DHA-treated cells clustered closely together. Hence, serum reduction and LPS treatment had a greater effect on the gene expression profile than DHA supplementation. Within each of the four main clusters, cells separated based on cell cycle phase, as assigned using the Seurat CellCycleScoring tool (**Figure S3C**).

**Figure 2:**
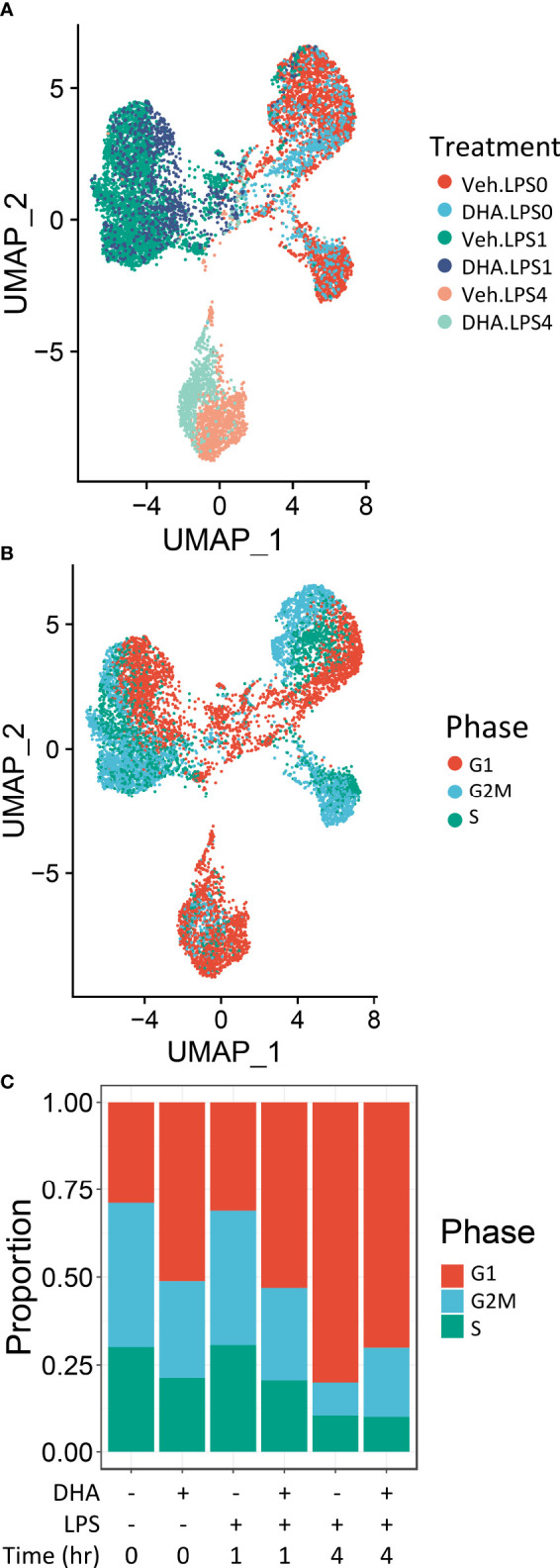
Cells cluster according to the presence and duration of LPS treatment. **(A)** Uniform manifold approximation and projection (UMAP) clustering revealed 3 distinct clusters of cells representing cells that received no LPS treatment cells treated for 1 h, and cells treated for 4h. **(B)** Distinct subclusters are present for cells in the G1, S and G2 phases of the cell cycle, as identified by Seurat CellCycleScoring function. **(C)** Treatment with DHA and with LPS influence the proportion of cells in each phase of the cell cycle.

Due to the robust transcriptional differences resulting from serum reduction and from cell cycle phase, we chose to create a new Seurat object including only FLMs cultured with reduced serum and where cell cycle genes were regressed out (**Figure 2A**). Cells still clustered based on cell cycle phase, but to a lesser extent than in the original Seurat object (**Figure 2B**). The number of cycling cells was reduced with DHA treatment in the absence of LPS and in the 1 h LPS treatment group. Treatment with LPS for 4 h substantially reduced the number of cycling cells (**Figure 2C**).

### FLMs retain macrophage-specific phenotype

**Figure 3:**
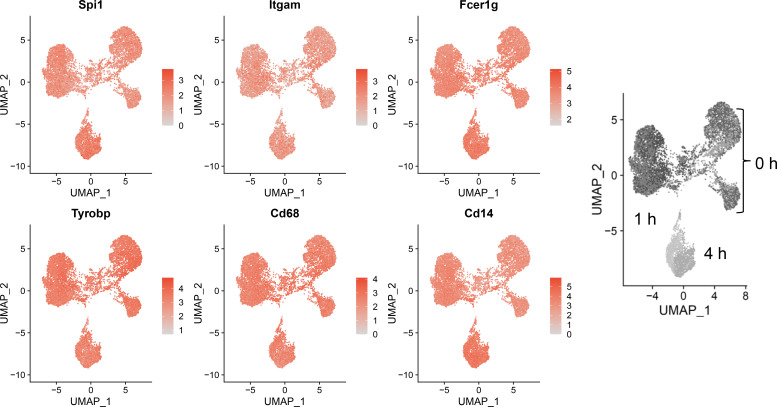
Self-renewing FLMs consistently express macrophage-specific genes across all times and treatments. Normalized expression values of individual macrophage-specific genes are overlayed on all cells are plotted using the Seurat FeaturePlot function. Macrophage-identifying genes are present in most cells in all time and treatment clusters indicating robustness of the self-renewing FLM model. Gray-scale image on right derived from (**Figure 2A**) indicates timepoints represented in clusters.

Sequencing data confirmed expression of macrophage-identifying genes in most cells in all clusters indicating that the FLM model maintained a macrophage-specific phenotype (**Figure 3**). Highly prominent was lineage-determining transcription factor PU.1 (*Spi1*), which is critical in macrophage and monocyte development (32). PU.1 is considered to be a pioneer transcription factor that binds to closed chromatin and prime promoter-distal enhancers. Other macrophage-defining genes that were similarly expressed strongly across all clusters included *Itgam*, *Fcer1g*, *Tyrobp*, *Cd68*, and *Cd14* (33–35). The demonstration that macrophage-identifying genes are present in most cells across all time and treatment clusters supports the contention that self-renewing FLMs are a robust macrophage model.

### DHA influences expression of genes involved in the antioxidant response, lipid and cholesterol metabolism, proliferation, metal homeostasis, and immune modulation at 0 h

**Figure 4:**
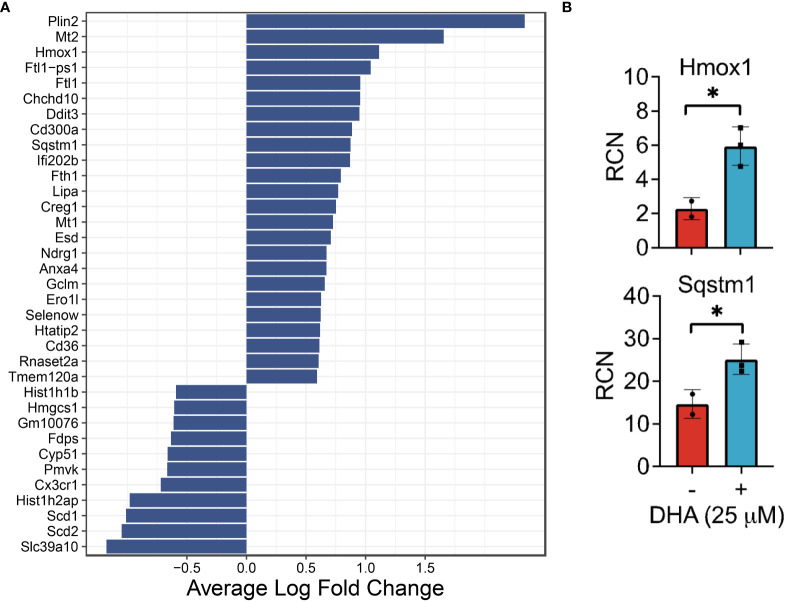
DHA influences expression of genes in FLMs involved in lipid uptake/metabolism (*Cd36, Plin2, Lipa*), antioxidant response (*Sqstm1, Hmox1, Chchd10)*, metal homeostasis (*Mt1, Mt2, Ftl1, Fth1)* and immune regulation (*Ifi202b, Cd300a, Anxa4*) at 0h. **(A)** Differentially expressed genes were identified using the Seurat FindMarkers function, with thresholds set to select genes with >1.5-fold change (Adjusted *p*-value < 0.001) in gene expression and genes expressed in at least half in the Veh.LPS.0 or DHA.LPS.0 groups. **(B)** Expression of *Hmox1* and *Sqstm1*, two genes induced by DHA treatment, was confirmed using bulk qPCR of samples treated in parallel with treatments for single cell isolation. Asterisks indicated significant differences *p<0.05) between Veh (-) and DHA (+) groups, as assessed by Student’s t-tests.

We identified 35 differentially expressed genes in the DHA treatment group compared to the Veh treatment group at 0 h (no LPS treatment) (**Figure 4A**). Of these, 11 were downregulated and 24 were upregulated. GO analysis only returned pathways that overlapped with 2 or 3 of the differently expressed genes, which made it difficult to determine the relevance of these results. We therefore chose to forego the GO analysis and instead performed literature searches to identify the cellular pathways involved with the differentially expressed genes.

DHA augmented expression of multiple genes that play roles in the antioxidant response and known to be induced by NRF2, including *Cd36*, *Sqstm1, Hmox1, Ftl1, Fth1*, and *Gclm* (36, 37). Bulk qPCR confirmed expression of *Hmox1* and *Sqstm1*, which encode the proteins heme-oxygenase-1 and sequestisome-1 and play roles in reducing reactive oxygen species and promoting autophagosome formation, respectively (38, 39) (**Figure 4B**). Augmented expression of NRF2-mediated genes is consistent with findings in other *in vitro* macrophage models and in preclinical animal studies employing omega-3 PUFA supplementation or endogenous omega-3 PUFA production promoted by expression of the *Fat1* transgene (40–44). Many NRF2 targets are important for detoxifying and countering oxidative stress within the cell, thus activation of this pathway has been posited as a key mechanism by which omega-3 PUFAs counter inflammatory stimuli. This mechanism is believed to involve ROS-induced non-enzymatic omega-3 PUFA oxidation products which form adducts with Kelch-like ECH-associated protein 1 (KEAP1) (45). While unmodified KEAP1 sequesters NRF2 in the cytoplasm, its adduction permits release of NRF2 into the cytoplasm thereby promoting expression of antioxidant genes. Thus, FLMs could be used in the future to track intracellular movement of KEAP1 and NRF2 in the presence and absence of DHA and other omega-3 PUFAs.

DHA also augmented genes involved in lipid uptake and synthesis, including *Cd36* (a scavenger receptor involved in lipid uptake)*, Plin2* (associated with lipid droplet formation), and *Lipa* (lipase involved in lysosomal degradation of lipids) (**Figure 4A**). In contrast, DHA-treated cells had lower expression levels of genes involved in cholesterol synthesis (*Scd1, Scd2*, *Pmvk, Cyp51, Hmgcs1, Fdps*). Changes to cholesterol synthesis may be in response to changes to membrane fluidity that occur with omega-3 PUFA incorporation into phospholipids (46).

It was further notable that DHA reduced expression of histone genes (*Hist1h1b, Hist1h2ap*), which is associated with a decline in DNA synthesis (47), and increased the apoptotic transcription factor *Ddit3*, which is consistent with the observed decrease in cycling cells (**Figure 4A**). Previous studies have shown that treatment with high concentrations of DHA may induce apoptosis *in vitro* in breast cancer cells, likely through exaggerated activation of the pathways observed in this study (48). We conclude that, in our model, while DHA treatment did reduce cell proliferation, the concentration was not high enough to trigger excess cell death by apoptosis.

DHA increased expression of genes associated with metal homeostasis (*Mt1*, *Mt2, Ftl1, Fth1*) (**Figure 4A**). Metallothionines (*Mt1, Mt2*) have been implicated in reducing inflammation in chronic inflammatory disease, but their mechanism of action is unclear (49). Ferritin light chain (*Ftl1*) has been shown to reduce NF-κB activation in Raw 264.7 macrophages (50).

Other differentially expressed genes (**Figure 4A**) have functions that are less well-described, though many are implicated in pathways involved in oxidative stress, cell proliferation, and the immune response. For example, it has been shown that mutations in *Chchd10* are associated with impaired mitochondrial genome maintenance following oxidative stress (51). *Ifi202b* (also known as p202) suppresses IFN gene signaling by binding to dsDNA and preventing its access to nucleic acid sensors in the cell (52). *CD300a* is an inhibitory receptor expressed on many immune cell populations (53). *Ndrg1* acts as a tumor suppressor in some cancers and a tumor promoter in others (54). We also observed increased expression of *Anxa4*, which was shown to inhibit adenylate cyclase 5 (55) and to be upregulated in human and mouse M2-polarized macrophages *in vitro* (56).

Altogether, the DHA-mediated changes in gene expression observed at 0 h are consistent with a putative shift in macrophage phenotype that would make FLMs more resilient against subsequent inflammatory triggers.

### LPS-induced cellular pathways promoting transcription precede inflammatory gene expression

**Figure 5:**
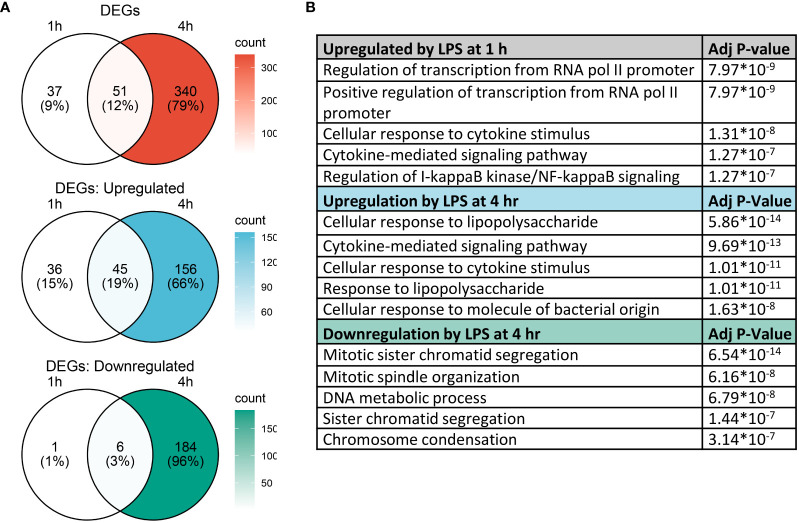
Gene expression changes induced by LPS in FLMs at 1 and 4h. **(A)** Differentially expressed genes (DEGs) were identified using the Seurat FindMarkers function, with thresholds set to select genes with >2-fold change in gene expression and genes expressed in at least half of either group being compared. Venn diagrams of DEGs at 1h only (left circle), 4h only (right circle), and at both timepoints (intersection) were generated to visualize the quantity of DEGs in each group. **(B)** The Enrichr database was used to identify GO Biological Process terms for the indicated groups of genes. Shown are the five most enriched pathways upregulated by LPS after 1 and 4 h or downregulated by LPS after 4 h, as determined by the size of the adjusted *p*-value.

Analysis with Seurat FindMarkers revealed 88 differentially expressed genes (>2-fold) after 1 h LPS treatment and 391 differentially expressed genes (>2-fold) with 4 h LPS treatment, with 51 genes shared between the two timepoints (**Figure 5A**). Among the genes differentially expressed at 1 h, 81 were upregulated and 7 were downregulated; and among the genes differentially expressed at 4 h, 201 were upregulated and 190 were downregulated.

We used the Enrichr database (57) to identify GO Biological Process terms associated with differentially expressed genes (**Figure 5B**). Genes upregulated at 1 h were enriched in pathways involved in regulating transcription as well as inflammatory pathways, like cellular response to cytokine stimulus and response to lipopolysaccharide. This suggests that at 1 h, many genes were setting the stage for the more robust transcriptional response subsequently observed at 4 h. Indeed, at 4 h, most upregulated genes are involved in inflammatory signaling pathways, while downregulated genes are involved in cell proliferation.

**Figure 6:**
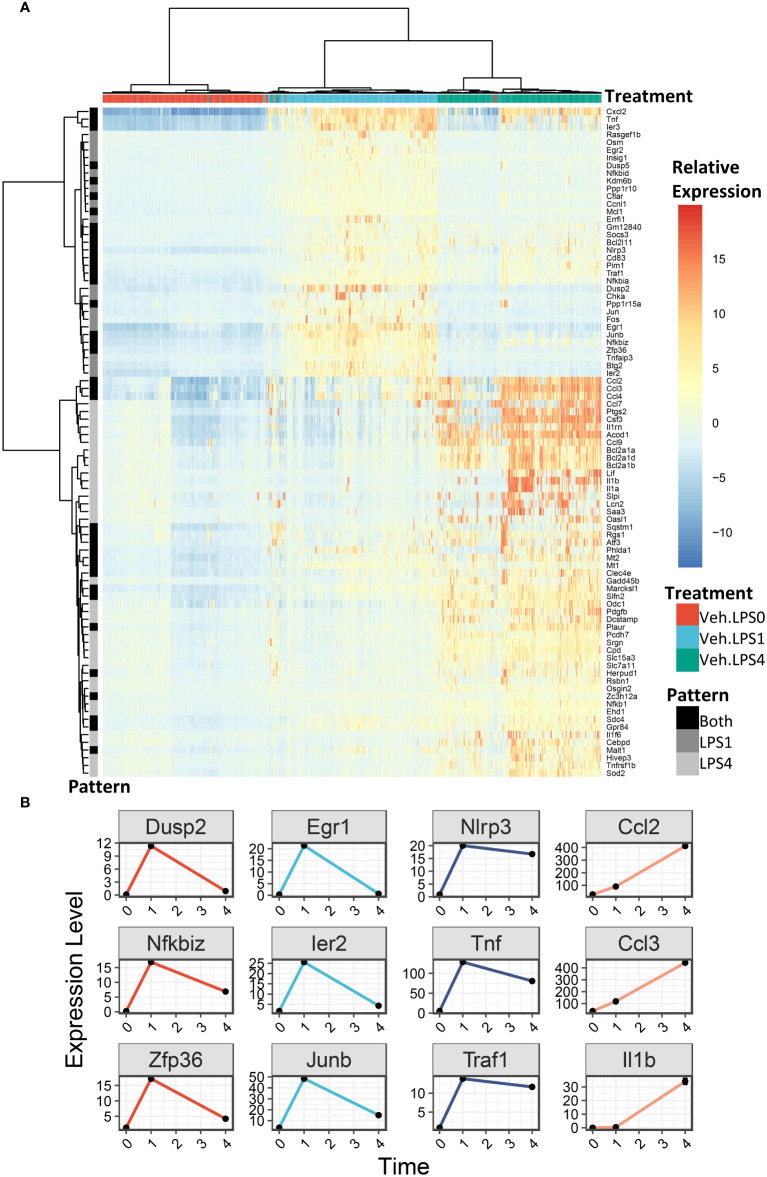
LPS robustly upregulates gene expression in FLMs after 1 and 4h. **(A)** 500 cells were randomly selected from the Veh.0, Veh.LPS.1, and Veh.LPS.4 groups and genes upregulated >6-fold were used to generate a heatmap using pheatmap with the “ward.d” clustering method. The normalized SCT values were plotted without scaling the heatmap. Cells were annotated according to their treatment group. **(B)** Plots showing the expression of individual LPS-induced genes at the 0, 1, and 4 h timepoint.

We generated heatmaps to visualize the expression pattern among individual cells for genes upregulated by LPS (**Figure 6A**, additional details in figure legend). Cells were annotated by treatment (colored horizontal lines above heatmap). As in the UMAP plots, unbiased clustering of cells resulted in near-perfect separation by LPS treatment group. Genes for which expression decreased from 1 h to 4 h post-LPS treatment include those involved in negative feedback of inflammatory signaling (*Dusp2, Nfkbiz, Zfp36*), transcription factors (*Egr1, Ier2, Junb*), and genes involved in promoting inflammatory signaling (*Nlrp3, Tnf, Traf*) (**Figures 6A, B**). Many other genes induced at 4 h have low or no expression at 1 h and code for cytokines or were proteins involved in the inflammatory response, such as *Ccl2* (MCP-1)*, Ccl3* (MIP-1a), and *Il1b* (IL-1β).

### DHA suppresses genes involved in cholesterol metabolism at 1 h post-LPS exposure

**Figure 7:**
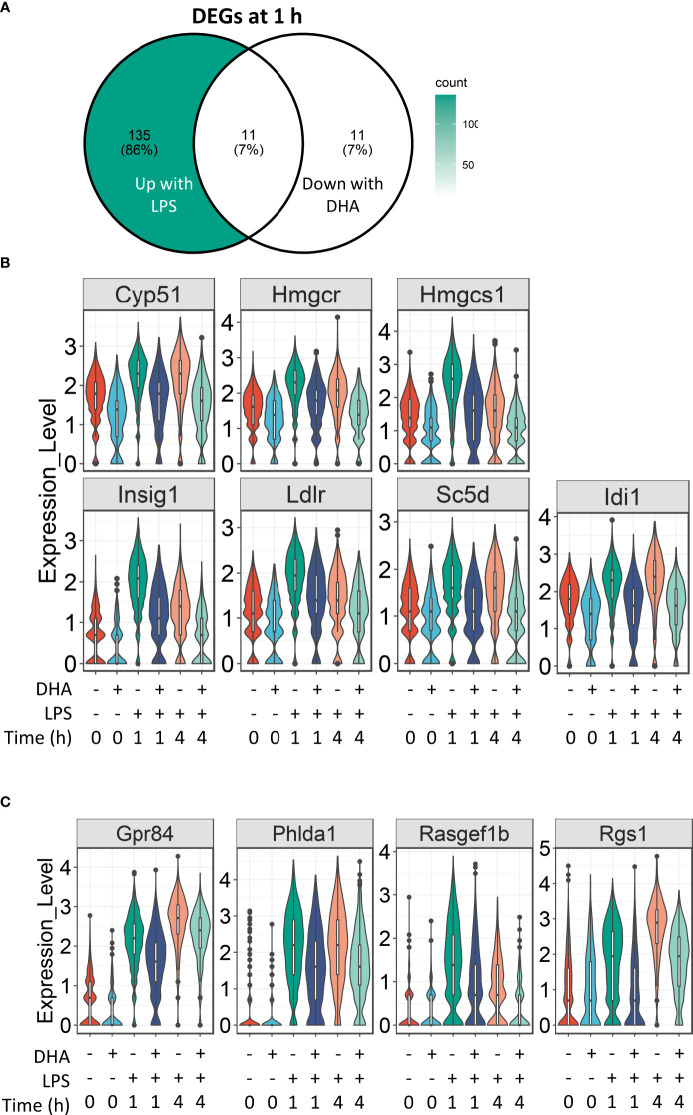
DHA suppresses expression of genes associated with cholesterol metabolism and inflammatory signaling pathways at 1 h post-LPS treatment. **(A)** Differentially expressed genes (DEGs) were identified by using the Seurat FindMarkers function, with thresholds set to select genes with >1.5-fold change in gene expression and genes expressed in at least 25% of groups being compared. DEGs in the “Down with DHA” circle of the Venn diagram are downregulated in DHA.LPS.1 relative to Veh.LPS.1 and DEGs in the “Up with LPS” circle of the Venn diagram are upregulated in Veh.LPS.1 relative to Veh. The intersection represents LPS-induced genes suppressed by DHA at 1h. **(B)** 7 of the 11 LPS-induced genes suppressed by DHA involve the cholesterol synthesis pathway. **(C)** Other LPS-induced genes suppressed by DHA at 1 h are also suppressed by DHA at 4 h.

For analysis of DHA effects at 1 h post-LPS treatment, we reduced the threshold for identifying differentially expressed genes to >1.5 fold to capture the nuanced effects of DHA more thoroughly. Following 1 h LPS treatment, 156 genes were induced and DHA significantly suppressed 11 of these (**Figure 7A**). More than half of the suppressed genes were involved in cholesterol synthesis, including *Cyp51, Hmgcs, Hmgcr, Insig, Ldlr, Sc5d*, and *Idi1* (**Figure 7B**). This is consistent with recent studies investigating the role of lipid metabolism in macrophages, where it has been shown that LPS-induced cholesterol synthesis promotes macrophage activation (58, 59). These findings are also consonant with our observation that DHA suppresses cholesterol synthesis genes in FLMs in the absence of inflammatory stimulus at 0 h (**Figure 4A**).

Our results suggest that DHA and LPS have opposing roles in cholesterol synthesis, revealing another potential mechanism by which DHA suppresses inflammatory signaling. While it is known that inhibition of cholesterol biosynthesis with statins can suppress inflammation, the specific effects on macrophages are less well understood (60). All the genes suppressed by DHA at 1 h post-LPS treatment are targets of the transcription factors SREBP1a and SRBP1c (61–63), which have been shown to be inhibited by DHA supplementation *in vitro* and *in vivo* (64, 65). An increase in intracellular cholesterol fosters TLR4 signaling by providing structure for lipid rafts, which promote aggregation of TLR4 receptor with its co-receptor CD14 (59, 66). Cholesterol depletion has also been shown to inhibit GPCR endocytosis (67). It is therefore tempting to speculate that this may also be the case for TLR4 endocytosis, which could explain decreased IRF signaling in DHA-treated cells (described further below).

The remaining genes induced after 1 h LPS treatment and suppressed by DHA are *Gpr84*, a GPCR known to be involved in activating inflammatory pathways in macrophages (68), *Phlda1*, which is highly expressed in inflammatory macrophages from atherosclerotic plaques (69), and *Rasgef1b* and *Rgs1*, which are involved in G-protein signaling (**Figure 7C**).

### DHA suppresses inflammatory signaling pathways at 4 h post-LPS exposure

**Figure 8:**
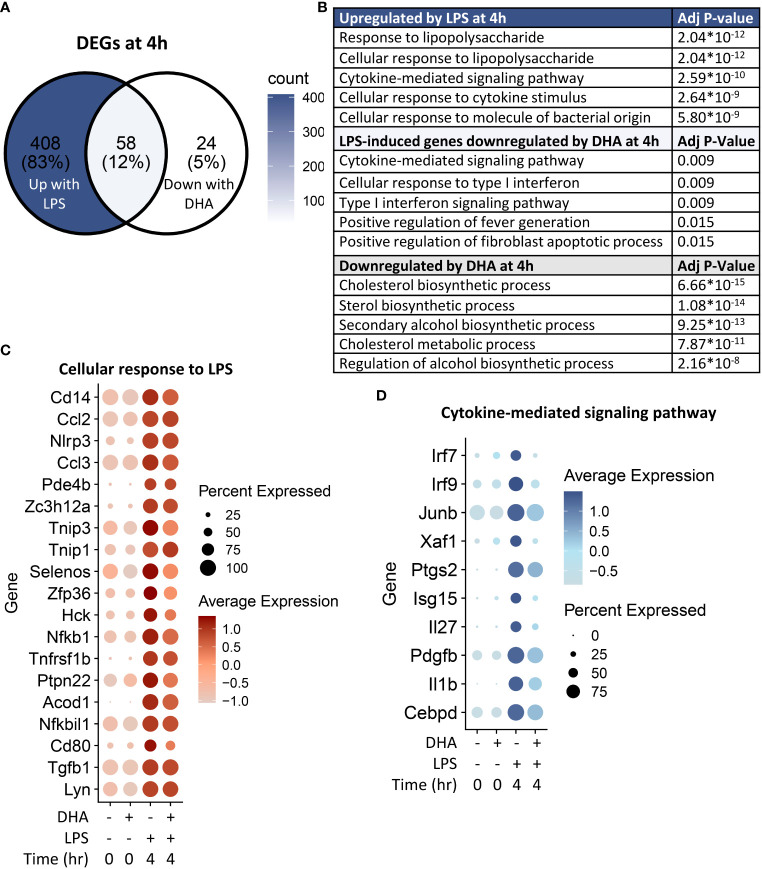
DHA inhibits inflammatory signaling pathways at 4 h post-LPS treatment. **(A)** Differentially expressed genes (DEGs) were identified by using the Seurat FindMarkers function, with thresholds set to select genes with >1.5-fold change in gene expression and genes expressed in at least 25% of groups being compared. DEGs in the “Down with DHA” circle of the Venn diagram are downregulated in DHA.LPS.4 relative to Veh.LPS.4 and DEGs in the “Up with LPS” circle of the Venn diagram are upregulated in Veh.LPS.4 relative to Veh. The intersection represents LPS-induced genes suppressed by DHA at 1h. **(B)** The Enrichr database was used to identify GO Biological Process terms for the indicated groups of genes. Shown are the five most enriched pathways are shown, as determined by the size of the adjusted *p*-value. **(C)** Selected input genes enriched in the “Cellular response to LPS” and **(D)** “Cytokine-mediated signaling pathway” GO terms are depicted using the Seurat DotPlot feature, where the size of the dot indicates the percent of cells expressing the gene and the depth of the dot color indicates the average expression of the gene across the cells in which it is expressed.

**Figure 9:**
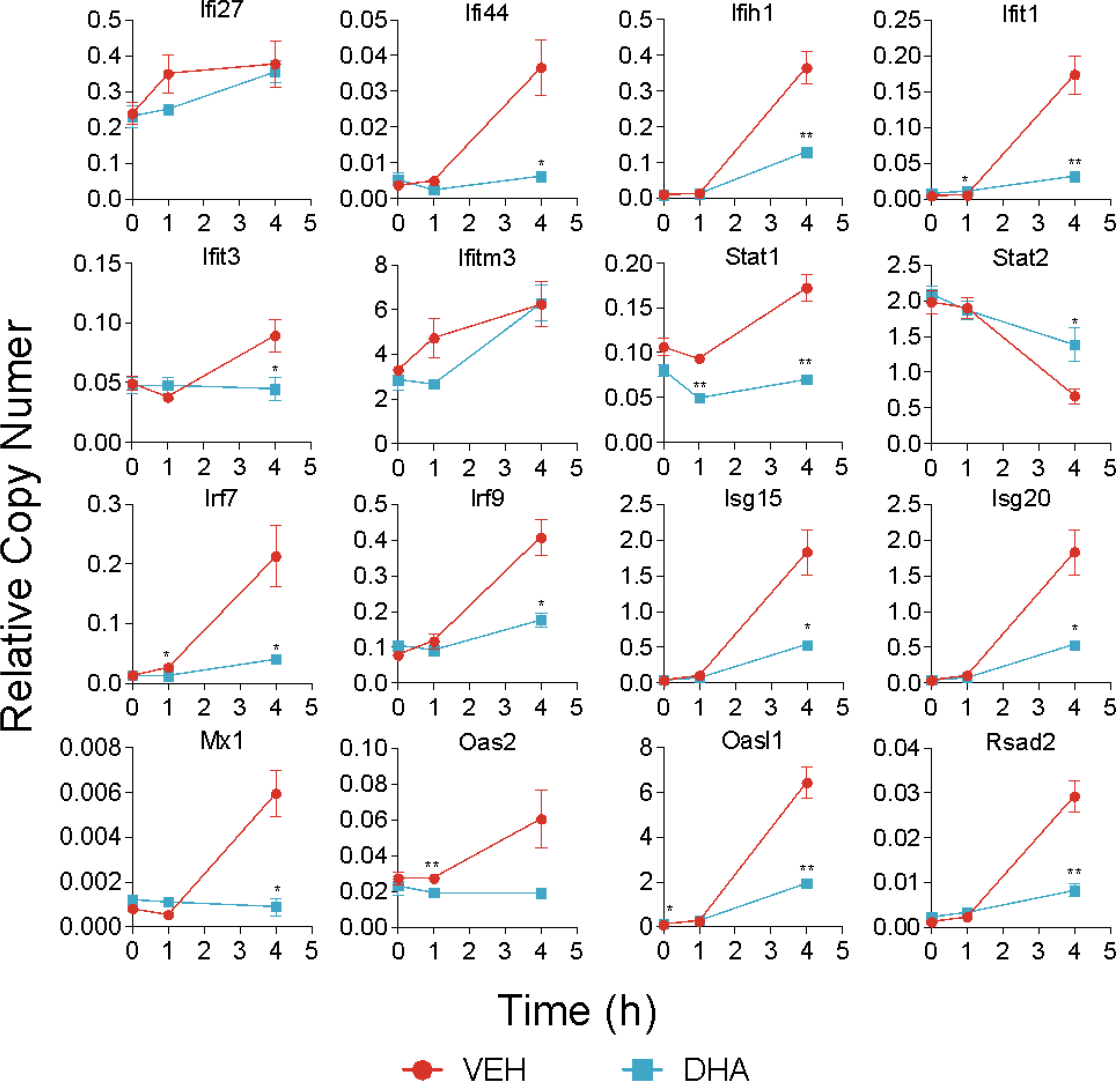
DHA suppresses expression of selected IFN1-regulated genes. A panel of IFN1-regulated genes was investigated by bulk qPCR in samples treated with LPS for 1 and 4 h. Cells were pre-treated with DHA or Veh for 24 h prior to LPS treatment. Asterisks indicate significant differences (*p<0.05, **p<0.01) between the DHA and Veh groups, n=3.

Following 4 h LPS treatment, 466 genes were induced by 1.5-fold or more (**Figure 8A**). Of the LPS-induced genes, 58 were suppressed by DHA (by at least 1.5-fold). We identified GO Biological Processes for the differentially expressed genes that were 1) increased by LPS, 2) increased in LPS and suppressed by DHA, and 3) decreased by DHA (**Figure 8B**). As observed at 1 h, DHA significantly suppressed many genes associated with cholesterol synthesis. LPS-induced genes that were suppressed by DHA were enriched for pathways such “cytokine mediated signaling pathway” and “cellular response to type I interferon”. Assessment of selected genes annotated as “cellular response to LPS” and “cytokine-mediated signaling pathway” show that DHA robustly suppressed certain genes while only mildly suppressing, or having no effect, on others (**Figures 8C, D**). The group of genes annotated as “cellular response to LPS” contained chemokines (*Ccl2, Ccl3*), cytokines (*Tgfb1*), and other genes promoting proinflammatory signaling pathways (*Nfkb1, Cd14, Tnfrsf1b, Nlrp3*). LPS also upregulates many genes that counteract inflammation by degrading mRNA coding inflammatory cytokines (*Zfp36*) or inhibiting inflammatory signaling pathways (*Tnip3, Tnip1, Nfkbil3*). Many of the genes annotated as “cytokine-mediated signaling pathway” are involved in or induced by IFN1 signaling (*Irf7, Irf9, Isg15, Xaf1*). We further confirmed DHA suppression of representative IFN1-regulated genes observed in scRNAseq using bulk qPCR (**Figure 9**).

### Distinct subsets of LPS-treated cells are driven by NF-κB and Irf signaling

**Figure 10:**
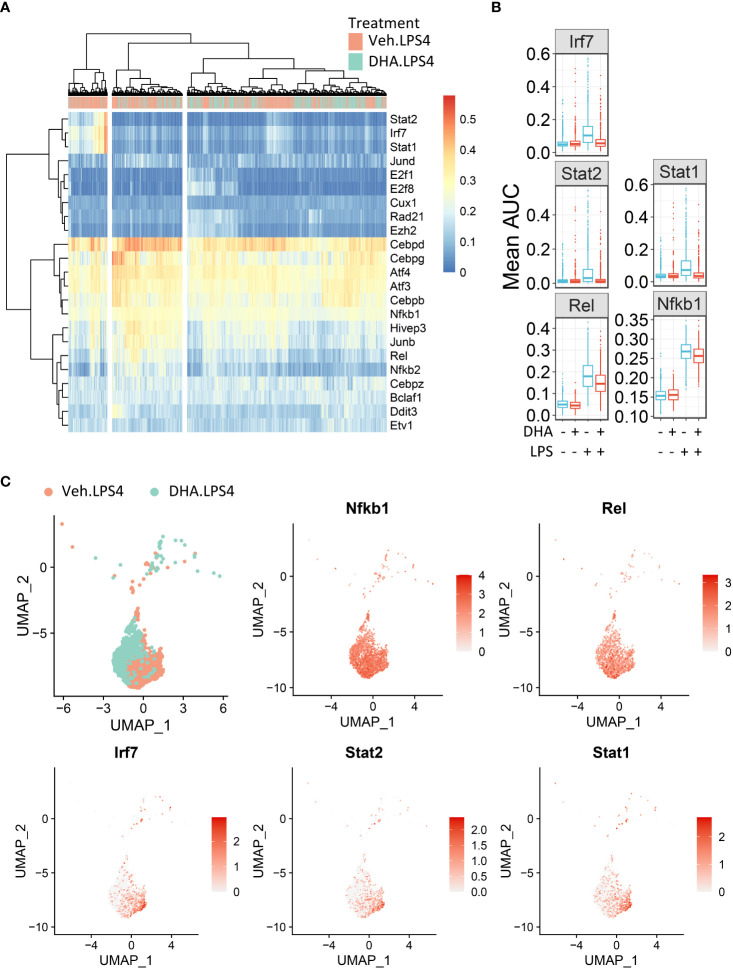
FLMs with high IFN1-regulated gene expression cluster together. **(A)** Regulon scores for cells in each group were generated using the SCENIC workflow. Regulons were selected that were significantly different between Veh.LPS.4 and DHA.LPS.4. Cells in the Veh.LPS.4 and DHA.LPS.4 treatment groups (as indicated by the horizontal bar above the heatmap) were analyzed for selected regulons. Clustering was performed using Ward’s method in the pheatmap tool in R Studio. The regulon AUC values were directly plotted without scaling the heatmap. **(B)** Boxplots for regulon scores were generated to identify differences between treatment groups. Irf7, Stat1, Stat2, NF-κB, and Rel regulons were increased by LPS at 4 hr. DHA reduced the regulon scores for NF-κB and Rel regulons and completely suppressed scores for Irf7, Stat1, and Stat2 regulons scores. **(C)** The Seurat FeaturePlot function was used to color cells from Veh.LPS.4 and DHA.LPS.4 treatment groups according to Irf7, Stat1, Stat2, Rel, and NF-κB1 regulon expression.

To identify in an unbiased manner which transcription factor programs (regulons) were most strongly induced by 4 h LPS treatment, we determined regulon scores for individual cells using the SCENIC workflow in R (27). Regulons consist of a group of co-expressed genes known to be induced by the same transcription factor, whose expression is also increased with expression of the target genes. Regulons that were significantly different between Veh and DHA at 4 h following LPS treatment, included transcription factors involved in NF-κB signaling (*Nfkb1* and *Rel*) and IFN signaling (*Irf7*, *Stat1*, and *Stat2*) (**Figure 10A**). The *Nfbk1* and *Rel* genes encode NF-κB family members p50 and c-Rel, respectively. The NF-κB p50 subunit dimerizes with the NF-κB p65 subunit, forming the most abundant of the Rel/NF-κB heterodimers (70, 71). DHA suppressed the Rel and NF-κB regulons at 4 h, though not completely (**Figure 10B**). Although the Irf7, Stat1, and Stat2 regulons were increased to a lesser extent than the Rel and NF-κB regulons, they were almost completely ablated by DHA. When investigating the regulon scores for individual cells, UMAP clustering identified that a cluster of cells with high expression of genes in the Irf7, Stat1, and Stat2 regulons among cells treated with LPS for 4h. This is distinct from the pattern of NF-κB and Rel expression, which is more homogenously expressed in LPS-treated cells (**Figure 10C**). Most cells in the region with the highest Irf7, Stat1, and Stat2 regulon scores were Veh.LPS cells, confirming that treatment with DHA inhibits the induction of these gene expression programs.

Previously published bulk mRNA studies described early, middle, and late time-dependent gene expression patterns in macrophages treated with LPS (7, 8, 72, 73). These studies assessed timepoints that extended beyond 4 h and found that many inflammatory cytokines and chemokines peak at 4 h whereas induction of most IFN regulated genes occurs slightly later. Differential activation of LPS-triggered genes can be explained by divergent signaling pathways induced by TLR4 activation, which have been comprehensively described (6, 74). Briefly, TLR4 activation induces signaling cascades initiating with the adapter protein MyD88 or the heterodimer TRIF/TRAM. MyD88-dependent signaling results in activation of the kinase transforming growth factor β-activated kinase 1 (TAK1) which phosphorylates IκB kinase (IKK) α/β. IKKα/β phosphorylates IκB. This frees the transcription factor NF-κB to translocate to the nucleus. Using Western blotting, we confirmed that LPS induced, and DHA suppressed this pathway in our FLM model (**Figure S4**).

**Figure 11:**
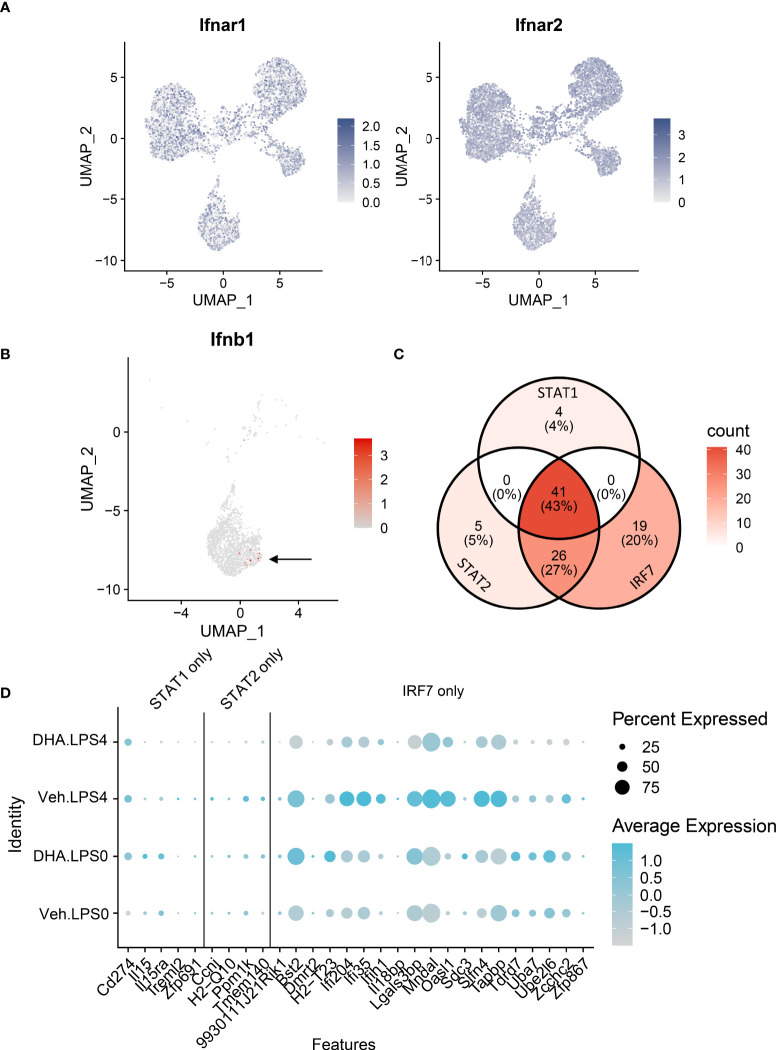
IFN1-regulated gene expression at 4 h post-LPS is driven by IRF rather than STAT activity. **(A)** *Ifnar1* and *Ifnar2* are homogenously expressed at a high level in the FLMs across the time clusters. **(B)** A small number of cells express *Ifnb* (arrow), and it is limited primarily to Veh-treated cells. **(C)** The majority of the genes identified in the STAT1 and STAT2 regulons are also IRF7 targets. **(D)** Genes driven by IRF7 alone are more highly expressed than genes driven by either STAT1 or STAT2 alone. Genes are presented using the Seurat DotPlot function, where the size of the dot indicates the percent of cells expressing the gene and the depth of the dot color indicates the average expression of the gene across the cells in which it is expressed.

The observations presented herein suggest that DHA impedes progression of TLR4-triggered IFN activation, a process that occurs more slowly than TLR4-triggered NF-κB activation. LPS binding can also induce endocytosis of the TLR4 receptor complex, resulting in a cascade of TRIF/TRAM and TBK1/IKKi activation, which phosphorylate IRF3 and IRF7. Phosphorylated IRF3 and IRF7 then translocate to the nucleus where they initiate I IFN1 signaling (75). Sheikh and coworkers (76) demonstrated that LPS-induced IFN1-regulated gene expression is critically dependent on intermediary IFN-β production and autocrine signaling through type I IFN receptors. Consistent with this possibility, we found *Ifnar1* and *Ifnar2* are homogenously expressed at a high level in the FLMs across the time clusters (**Figure 11A**) and that modest *Ifnb1* expression is detectable in some FLMs in the Veh/LPS 4 h group but not the DHA/LPS 4 h group (**Figure 11B**). The moderate response is likely due to the early timepoint at which the cells were collected and the limited sensitivity of scRNAseq for detecting low abundance transcripts. STAT1 and STAT2 are not directly activated by LPS, but instead are activated by IFNα/β receptor signaling. Though SCENIC predicted STAT1/2 activation, we conclude that this is due to the high degree of target gene overlap between these transcription factors and IRF7 (**Figure 11C**). Expression of genes predicted to be solely STAT1 or STAT2 is very low compared to IRF7-specific-target genes, supporting the argument that at 4 h LPS, IRF7 is the primary transcription factor driving this phenotype (**Figure 11D**). Thus, we predict that STAT1/2 activation would follow shortly after this timepoint due to release of IFNβ.

The observation that DHA suppresses TLR4-driven IFN1-regulated gene responses is of high translational relevance. Upregulated IFN1 responses contribute to the development of multiple forms of autoimmunity including lupus, systemic sclerosis, Sjogren’s syndrome (77). Indeed, successful clinical trials of anifrolumab, an anti-IFNAR monoclonal antibody (78), led to its approval by the USFDA in 2021 for lupus treatment (79). Relatedly, in a prior *in vivo* study, we observed that DHA supplementation significantly reduces IFN1-regulated gene expression in lupus-prone mice induced by exposure to the autoimmune trigger crystalline silica (80). It is particularly notable that many of the same IFN1-regulated genes identified *in vivo* were significantly enhanced here by LPS at 4 h and suppressed by DHA. Intriguingly, in both our *in vivo* and *in vitro* experiments, DHA suppresses the IFN1 pathway more strongly than other inflammatory pathways including NF-κB signaling. While Kobayashi and coworkers similarly found that the omega-3 fatty acids suppressed IFN1 gene expression in imiquimod (IMQ)-induced murine lupus (81), the underlying molecular mechanisms are unclear. Further investigations into how DHA preferentially suppresses could lead to novel therapies to effectively target this potent inflammatory response in autoimmune disease.

### Limitations

This exploratory study had several limitations that will necessitate additional expanded experimentation. First, extending the time window for LPS treatment could provide greater insight into the pathways most effectively targeted by DHA. Also, even though we identified that upstream inflammatory pathways (i.e., NF-κB signaling) were influenced by DHA supplementation, there is further need to confirm the effects on other key proteins associated with additional pathways identified, or on resultant metabolites produced (i.e., cholesterol metabolism). Furthermore, while the use of SCENIC analysis to identify regulons allows us to infer transcription factor activity based on concordant upregulation of the transcription factor and its putative target genes, this approach cannot prove activation of transcription factors of interest. Additional experiments would need to be performed to confirm the activation of NRF2, IRF7, STAT1, and STAT2. Finally, although the *in vitro* macrophage model used here is similar to an inflammatory monocyte-derived macrophage, it might not precisely mimic tissue-specific macrophage populations observed *in vivo*. Thus, many of the pathways identified here need to be confirmed in tissue-relevant models such as alveolar-like macrophages derived by culturing fetal liver cells with GM-CSF and TGF-β (82).

## Conclusions

**Figure 12:**
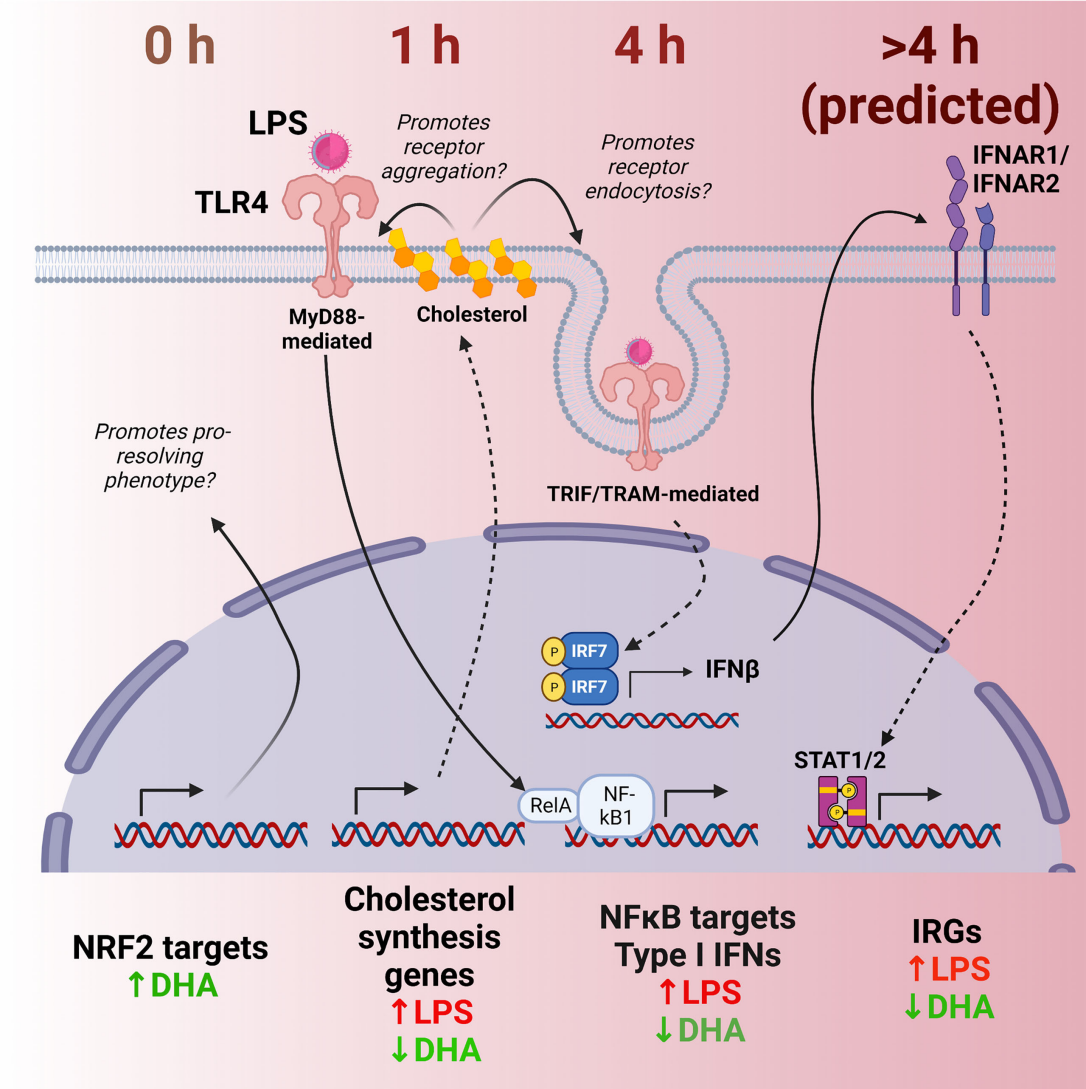
Putative model for DHA suppression of LPS-induced proinflammatory and IFN1-regulated gene expression in the macrophage. Time-dependent transcriptional changes elicited by DHA and/or LPS reveal multiple potential mechanisms by which DHA may counteract the inflammatory response to LPS. First, at 0 h (in the absence of LPS), DHA triggers expression of NRF2 target genes, which may promote a pro-resolving macrophage phenotype. At 1 h following LPS exposure, DHA suppresses LPS-triggered cholesterol genes, which may prevent cholesterol-mediated facilitation of further TLR4 signaling. At 4h following LPS exposure, DHA downregulates NF-κB-target and IFN1-regulated gene (IRGs) including IFN-β, likely preventing robust induction of further IFN-response at ≥ 4h.

As an innate immune population, macrophages act as sentinels to signal the presence of foreign molecules while simultaneously functioning to destroy or sequester invading microbes or injurious particles. Cytokines released by macrophages modulate phenotype and function of incoming immune cells, influencing the inflammatory response and setting the stage for an adaptive immune response. Sets of genes are upregulated in conjunction with each other at specified times, triggering a sequence of responses in the cell and extracellular milieu. Here we used scRNAseq to discern how DHA influences LPS-induced gene expression in FLMs, a primary, heterogeneous self-renewing macrophage model. This strategy allowed us to capture heterogeneity within a single population of treated cells, with groups of cells within the same LPS treatment group showing different patterns of inflammatory gene expression. As depicted in **Figure 12**, analyzing cells at different timepoints following LPS treatment permitted us to identify putative anti-inflammatory transcriptional mechanisms sequentially influenced by DHA in the absence of inflammatory stimuli (0 h), immediately following an inflammatory stimulus (1 h), and in the early stages of the inflammatory response (4 h). First, we found that DHA promoted basal antioxidant NRF2-target gene expression. Second, our analyses further uncovered cholesterol synthesis as a potential metabolic pathway differentially modulated by DHA and LPS that may be central to DHA-mediated suppression of TLR4-dependent signaling. Third, we observed that DHA suppresses IFN-driven genes in addition to NF-κB-driven genes, which provides a new perspective on how the multifaceted TLR4-induced signaling pathway is influenced by DHA. Employing scRNAseq enabled us to ascertain that IFN-driven genes are not homogenously expressed in a population of LPS-treated cells, which reveal a role for DHA in blocking or slowing cellular pathways that promote IFN signaling. Taken together, our findings herald lines of future research to investigate precision nutritional intervention with omega-3 PUFAs to combat chronic inflammation and progression of autoimmune disease.

## Data availability statement

The data presented in the study are deposited in the NCBI GEO repository, accession number GSE207902.

## Ethics statement

The protocol for FLM isolation was reviewed and approved by Michigan State University Institutional Animal Care and Use Committee.

## Author contributions

KW: initial study design, data analysis/interpretation, figure preparation, manuscript preparation, project funding. FR, BW: data curation, data analysis/interpretation, figure preparation, manuscript preparation. JH: initial study design, project funding, manuscript preparation. JP: initial study design, manuscript preparation, supervision, project funding. All authors contributed to the article and approved the submitted version.

## Funding

This research was funded by NIH ES027353 (JP), NIH F31ES030593 (KW), NIH T32ES007255 (KW), Lupus Foundation of America (JP, KW), USDA National Institute of Food and Agriculture Hatch Projects 1020129, (JP), the Dr. Robert and Carol Deibel Family Endowment (JP), ZonMW grant no. 91116011 (BW) and the Dr. Albert C. and Lois E. Dehn Endowment in Veterinary Medicine (JH).

## Acknowledgments

We would like to thank Anthony Bach and Dr. Norb Kaminiski for their assistance with isolation of single cells and preparation of Illumina sequencing libraries.

## Conflict of interest

The authors declare that the research was conducted in the absence of any commercial or financial relationships that could be construed as a potential conflict of interest.

## Publisher’s note

All claims expressed in this article are solely those of the authors and do not necessarily represent those of their affiliated organizations, or those of the publisher, the editors and the reviewers. Any product that may be evaluated in this article, or claim that may be made by its manufacturer, is not guaranteed or endorsed by the publisher.
