# Supplementary material for: Single cell analysis of docosahexaenoic acid suppression of sequential LPS-induced proinflammatory and interferon-regulated gene expression in the macrophage
Source: Front Immunol. 2022 Nov 3;13:993614. doi: 10.3389/fimmu.2022.993614 (PMC9669445; doi:10.3389/fimmu.2022.993614)
Supplement: Supplementary file 1 [file DataSheet_1.pdf]

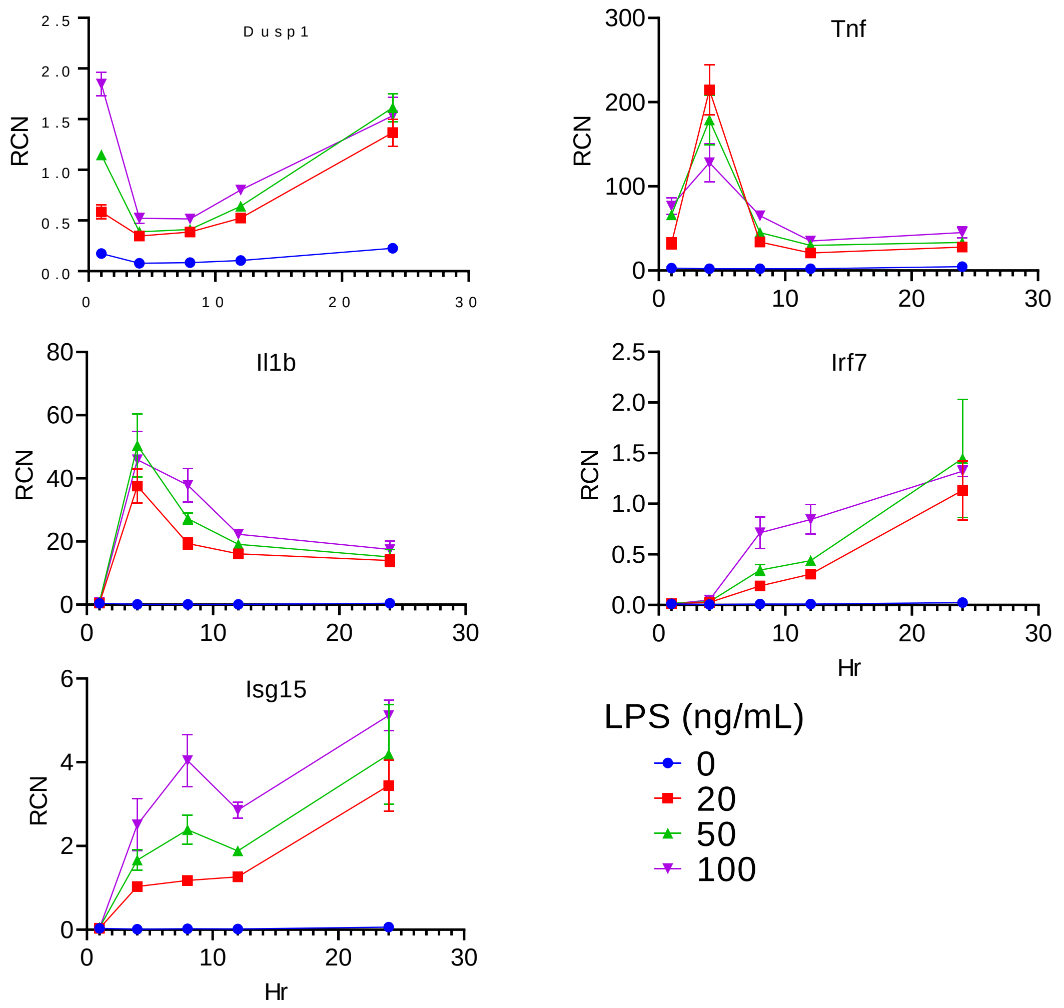

Figure S1. Preliminary LPS timecourse and dose response. FLMs were treated with LPS at the indicated doses and for the indicated times. Expression of inflammatory genes of interest was assessed by qPCR. n=3.

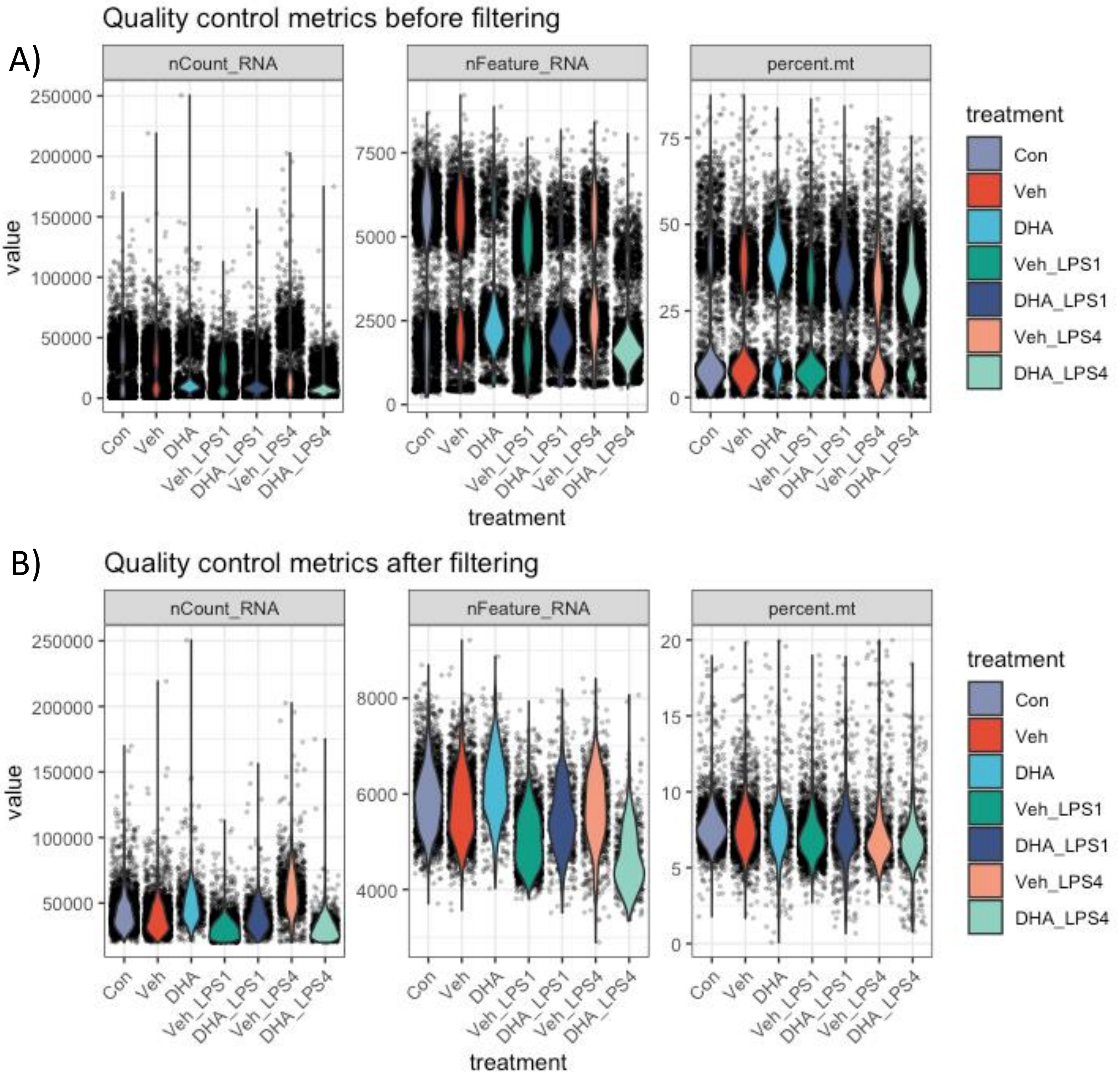

Figure S2. Quality control metrics before and after filtering. Only high-quality cells were used for downstream analysis. **(A)** Low quality cells were filtered out based on the presence of excess mitochondrial RNA (>20%) and low gene counts (nCount_RNA<20,000, nFeature_RNA <3000). **(B)** After filtering, each treatment group showed similar levels of the indicated quality control metrics.

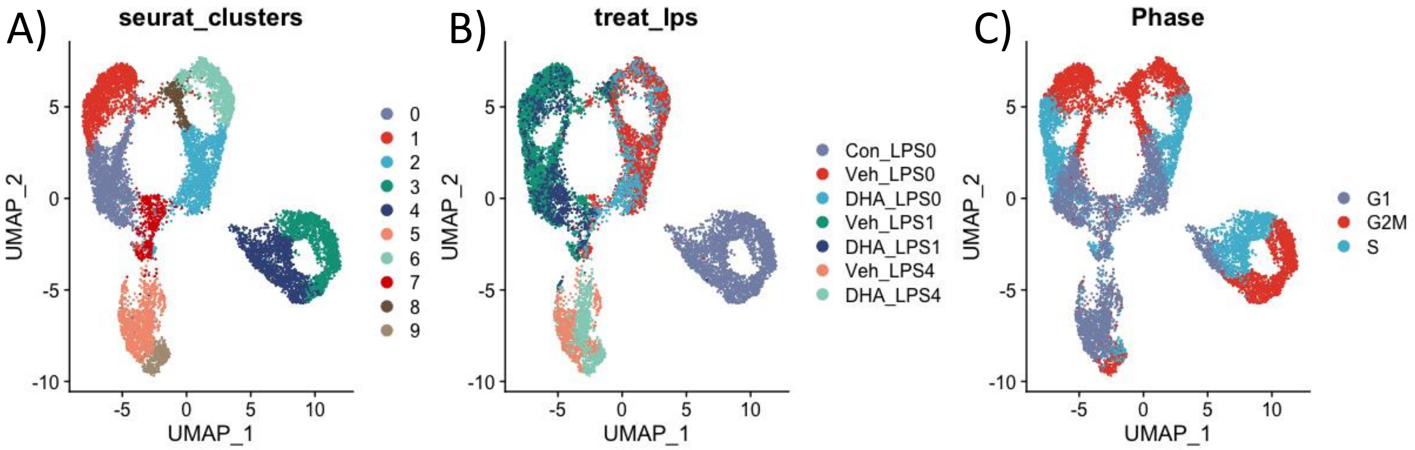

Figure S3. Uniform manifold approximation and projection (UMAP) reveals factors that drive clustering. (**A**) Unbiased Seurat Clustering identified ten distinct clusters which separated into four larger groups. (**B**) Labeling individual cells according to treatment showed that cells clustered into groups based on serum deprivation (-24 h vs 0, 1, and 4 h) and by duration of LPS treatment (0h vs 1h vs 4 h). (**C**) Cell cycle phase scores were applied to each cell using the Seurat CellCycleScoring function. Cells within the same phase clustered together within the groupings were separated based on serum deprivation and time of LPS treatment.

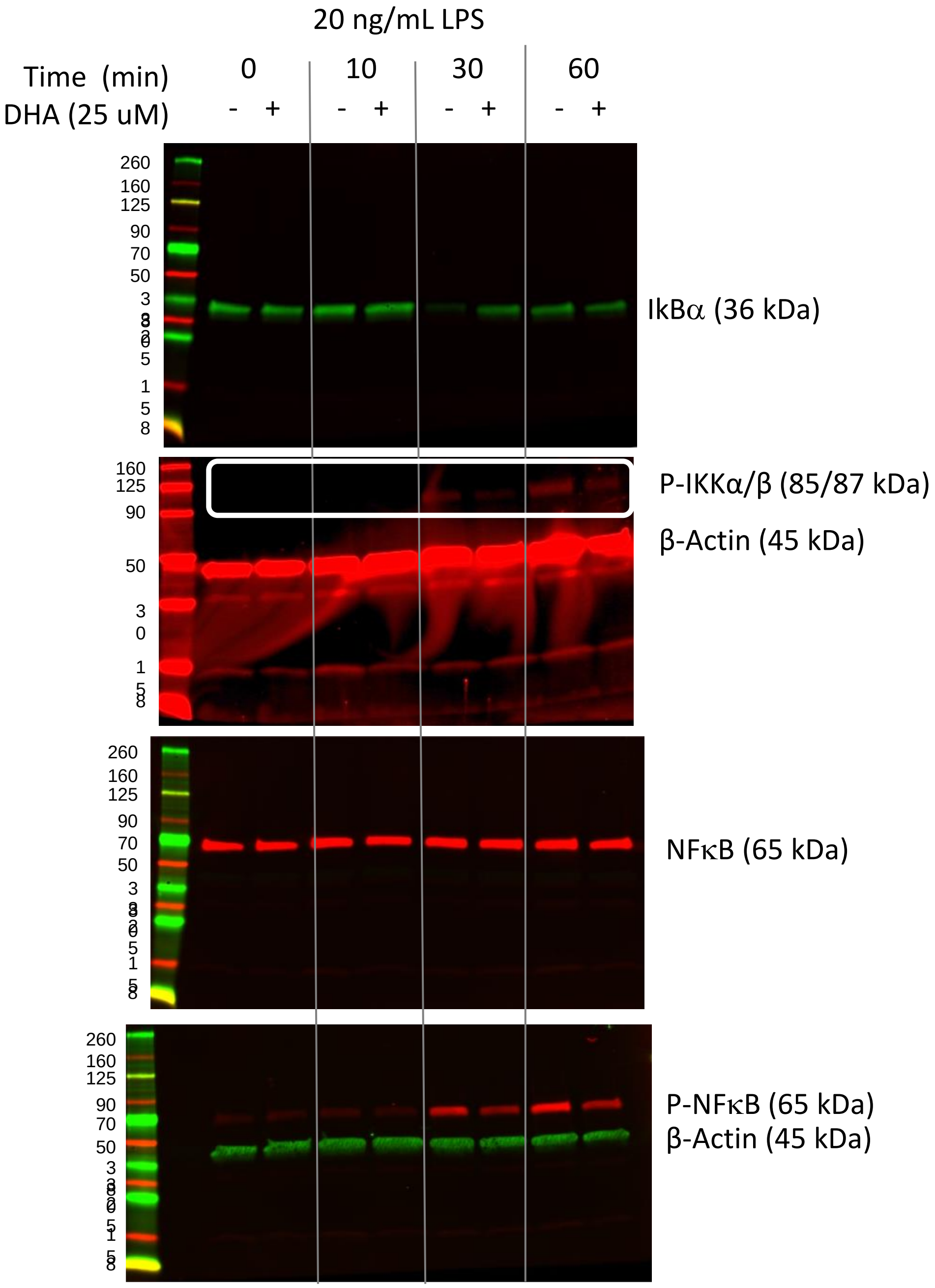

Figure S4. DHA inhibits NF-κB signaling pathway. FLM cells were treated with 20 ng/mL LPS and 25 μM DHA for the indicated times and cell lysates collected and processed for Western blots. Equal amounts of protein were loaded in each well of precast SDS-PAGE gels and proteins transferred to low-fluorescence nitrocellulose membranes. Membranes were probed for the indicated proteins and phospho-proteins and scanned using a Licor scanner. Actin and non-phosphorylated proteins were used as loading controls. Representative of 3 independent experiments.
